# Balloon assisted, ultrasound guided percutaneous thrombin injection of a large radial artery pseudoaneurysm using a trans-venous approach via an Ipsilateral Arteriovenous Fistula

**DOI:** 10.1186/s42155-021-00253-3

**Published:** 2021-08-26

**Authors:** Mark Gregory, Mike Guest, Islam Abdeen, Kate Steiner

**Affiliations:** 1 Department of Radiology, East and North Hertfordshire NHS Trust, Coreys Mill Lane, SG1 4AB Stevenage, UK; 2 Department of Vascular Surgery, East and North Hertfordshire NHS Trust, Coreys Mill Lane, SG1 4AB Stevenage, UK

**Keywords:** Pseudoaneurysm, Thrombin, Dialysis access, Arteriovenous Fistula

## Abstract

**Background:**

Pseudoaneurysm formation is known to complicate arteriovenous haemodialysis access. Ultrasound guided thrombin injection is a recognised treatment option, but is not possible in pseudoaneurysms with no measurable neck. Balloon assisted techniques have been described in such cases, which transiently obstruct flow out of the pseudoaneurysm and thereby prevent non-target embolization during ultrasound guided percutaneous thrombin injection. We describe a balloon assisted technique for the treatment of a radial artery pseudoaneurysm, via retrograde access from the draining cephalic vein of an arteriovenous fistula.

**Method:**

A 61-year-old male with a radio-cephalic fistula was found on duplex ultrasound to have a large radial artery pseudoaneurysm with no measurable neck, as well as a juxta-anastomotic cephalic vein stenosis. Endovascular treatment was selected over open surgery. Retrograde cephalic venous access was established, which allowed for concurrent treatment of both the venous stenosis and the arterial pseudoaneurysm. After balloon dilation of the juxta-anastomotic stenosis, a percutaneous transluminal angioplasty balloon catheter was advanced across the arteriovenous anastomosis and inflated across the neck of the radial artery pseudoaneurysm, to transiently obstruct blood flow. This allowed for safe injection of thrombin into the pseudoaneurysm by direct ultrasound guided sac puncture; thereby achieving thrombosis.

**Conclusions:**

Balloon assisted ultrasound guided thrombin injection is an endovascular treatment option that can obviate the need for open surgery in cases involving pseudoaneurysms with no measurable neck. The technique described allowed both concurrent treatment of a juxta-anastomotic venous stenosis and treatment of an arterial pseudoaneurysm from a single venous puncture. This technique avoided arterial access and its inherent complications.

## Background

Pseudoaneurysm formation is a recognised complication affecting arteriovenous haemodialysis access. Cases that are not suitable for open surgical repair are often treated with minimally invasive techniques including ultrasound guided thrombin injection (UGTI) (Corso et al. 2006) and endovascular stent graft placement (Wong et al. 2016). Stent grafts can be complicated by infections and stent migration (Dennis Wright et al. 2013), whereas UGTI is indicated only in pseudoaneurysms with a suitable neck to minimise the risk of thrombin embolization into the systemic circulation (Vowels et al. 2020).

Endovascular, balloon-assisted techniques transiently reduce blood flow across wide necked pseudoaneurysms, facilitating safe UGTI. This technique is usually employed for femoral artery pseudoaneurysms (Vowels et al. 2020), but may also be used to treat pseudoaneurysms complicating dialysis access (Mary et al. 2020; Clark and Abraham 2000).

We describe the treatment of a radial artery pseudoaneurysm in a patient with a radio-cephalic arteriovenous fistula (AVF), using balloon-assisted UGTI with retrograde access from the cephalic vein draining the AVF. The procedure was undertaken on an outpatient basis, concurrently with venoplasty to treat a juxta-anastomotic cephalic vein stenosis.

## Main Text

**Fig. 1:**
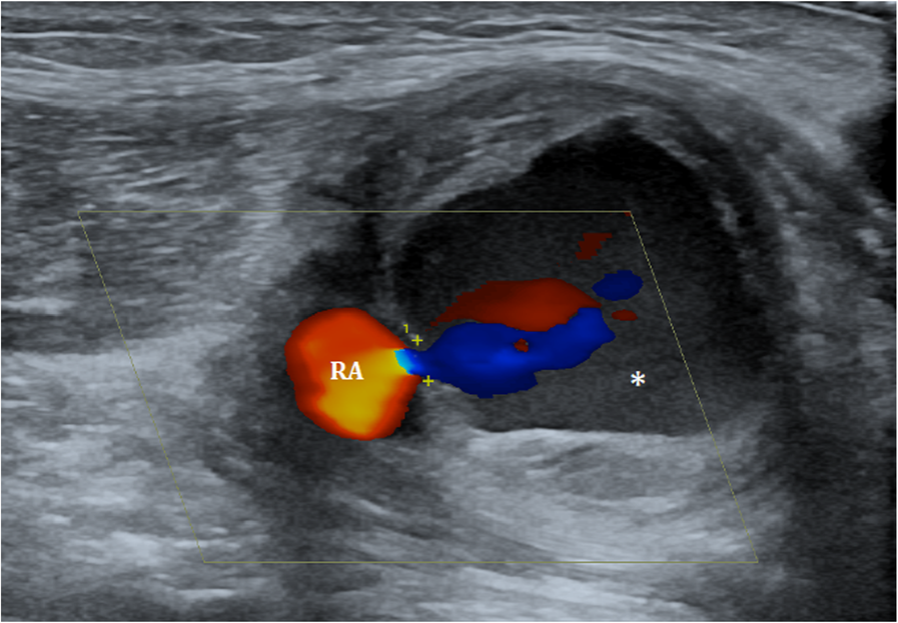
Duplex ultrasound image of the pseudoaneurysm (*) arising from the radial artery (RA), in the transverse plane. The neck of the pseudoaneurysm (+) has no measurable length.

A 61-year-old male with a left radio-cephalic fistula presented with a low haemodialysis flow rate of 270ml/min; measured using a Transonic® flow-QC device (Transonic Systems Inc. Ithaca, USA). Duplex ultrasound (DUS) demonstrated a 24mm diameter pseudoaneurysm arising directly from the radial artery. Whilst the neck of the pseudoaneurysm was narrow, there was no measurable neck length, increasing the risk of non-target embolization with standard thrombin injection techniques (Fig. 1).

**Fig. 2:**
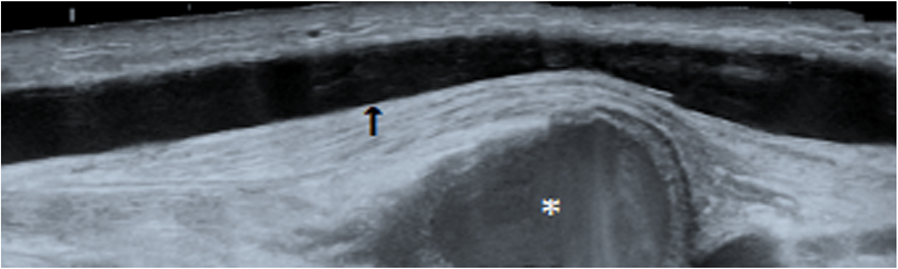
B-Mode ultrasound image of the cephalic vein in the longitudinal plane (black arrow). The pseudoaneurysm (*) is demonstrated lying deep to the cephalic vein, directly in line with the needling site.

The pseudoaneurysm was positioned deep to the cephalic vein; directly in line with the needling site (Fig. 2). DUS also identified a juxta-anastomotic cephalic vein stenosis. A decision was made to treat the pseudoaneurysm with balloon assisted UGTI, after discussion of the alternative surgical options with the Vascular Surgery team at a multidisciplinary team meeting.

An ultrasound guided retrograde cephalic vein puncture was performed and a short 5 F sheath inserted. Heparin 3,000 units was administered. A 0.018in Terumo Glidewire Advantage and a 4 F Cordis C2 catheter were advanced across the anastomosis into the proximal radial artery. Catheter angiography demonstrated the juxta-anastomotic stenosis. Following exchange for a V18 wire, the stenosis was treated with a 6mm x 40mm Mustang Balloon (Boston Scientific), with good angiographic result.

A 7 × 40mm Sterling Balloon (Boston Scientific) was then advanced over the wire and positioned across the neck of the pseudoaneurysm under ultrasound guidance. The balloon was sized to match the diameter of the radial artery, which measured 7mm on DUS. The pseudoaneurysm was punctured with a 21-guage needle under ultrasound guidance. The 7 × 40mm Balloon was then inflated, covering the pseudoaneurysm neck. 2mls of Thrombin was injected under ultrasound guidance, filling the pseudoaneurysm. The balloon was then deflated.

After balloon deflation, DUS demonstrated a small amount of residual perfusion within the largely thrombosed pseudoaneurysm sac. This appeared to have resolved following ultrasound guided manual compression of the pseudoaneurysm. Follow up DUS 72 h later demonstrated a persistent 8mm area of perfusion within the pseudoaneurysm. A repeat procedure was therefore undertaken, employing the same technique as the initial procedure, requiring a further 1mls of thrombin to achieve complete thrombosis.

**Fig. 3:**
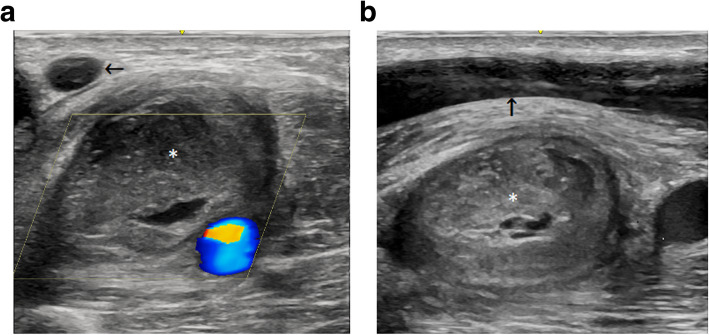
**a**. Duplex ultrasound image of the thrombosed pseudoaneurysm (*) in the transverse plane. The cephalic vein (black arrow) and the radial artery are also demonstrated. **b**. B-Mode ultrasound image of the thrombosed pseudoaneurysm (*) in the longitudinal view. The cephalic vein (black arrow) and the radial artery are also demonstrated.

No complications occurred during or immediately after either procedure. Both procedures were undertaken as day cases. A follow up ultrasound scan 72 h after the second procedure demonstrated complete thrombosis of the pseudoaneurysm with volume flow of 1400mls/min recorded in the brachial artery (Fig. 3). Successful two-needle cannulation of the fistula was achieved during future haemodialysis sessions.

## Discussion

We present a case of a radial artery pseudoaneurysm treated with balloon assisted UGTI with retrograde venous access via an ipsilateral radio-cephalic AVF. Whilst balloon assisted techniques for pseudoaneurysm embolization have been described previously (Mary et al. 2020; Clark and Abraham 2000), this case describes a variation of the technique which is of relevance to the dialysis population. Our case demonstrates the feasibility of using balloon assisted UGTI to treat pseudoaneurysms with no measurable neck.

Our patient presented due to low haemodialysis flow and it is likely that resultant venepuncture difficulties caused the pseudoaneurysm, which lay immediately deep to the venous needling site. The position of the pseudoaneurysm also left it susceptible to further injury from inadvertent puncture during subsequent AVF cannulation.

The location of the pseudoaneurysm in the mid/upper forearm made open surgery a technically difficult, higher risk treatment option. The radial artery lies deep to pronator teres at this position and the draining cephalic vein directly overlying the pseudoaneurysm would have needed to be mobilised. The patient also wished to avoid an overnight admission due to social circumstances.

Treatment with a covered stent was considered and would have been attempted had balloon-assisted thrombin injection failed. The endovascular approach via the draining vein of the AVF also allowed concomitant treatment of the juxta-anastomotic stenosis; improving flow within the fistula.

Arterial pseudoaneurysms in patients with radio-cephalic AVFs have previously been treated with balloon assisted techniques via a retrograde puncture of the distal radial artery (Aytekin et al. 2002). The distal radial artery in this case branched after a low AVF anastomosis and was not suitable for percutaneous access, especially if a 7Fr sheath had been required for covered stent insertion. Percutaneous puncture of the draining vein of an AVF is associated with a low risk of puncture site complications, even when using larger sheaths required for stent graft insertion. Puncture of the ipsilateral brachial artery would have been the alternative approach, carrying a higher risk of puncture site complications.

Residual filling of the pseudoaneurysm was identified by DUS following the first attempt at Thrombin injection and a second procedure was required to achieve sustained thrombosis. DUS follow up post procedure is essential to ensure thrombosis with no re-perfusion. At the time of writing the patient continues to dialyse successfully with no further complications. Clinical surveillance continues with physical examination of the AVF and regular measurement of volume flow on dialysis.

## Conclusions

This case demonstrates the feasibility of treating AVF related arterial pseudoaneurysms with no measurable neck with balloon assisted UGTI. Venous access in these cases may be preferable as it allows for concurrent treatment of AVF stenoses and carries a low risk of puncture site complications. The technique is adaptable for different anatomy and type of AVF and offers a minimally invasive treatment option as an alternative to open surgery.

## Data Availability

Not applicable.

## Abbreviations

AVF: Arteriovenous Fistula.
DUS: Duplex Ultrasound.
UGTI: Ultrasound Guided Thrombin Injection.
